# Telerehabilitation and Wellbeing Experience in Children with Special Needs during the COVID-19 Pandemic

**DOI:** 10.3390/children8110988

**Published:** 2021-11-01

**Authors:** Daniela Sarti, Marinella De Salvatore, Emanuela Pagliano, Elisa Granocchio, Daniela Traficante, Elisabetta Lombardi

**Affiliations:** 1 Foundation IRCCS Neurological Institute ‘C. Besta’, 20133 Milan, Italy; daniela.sarti@istituto-besta.it (D.S.); marinella.desalvatore@istituto-besta.it (M.D.S.); emanuela.pagliano@istituto-besta.it (E.P.); elisa.granocchio@istituto-besta.it (E.G.); 2 Department of Psychology, Catholic University of Sacred Heart, 20123 Milan, Italy; daniela.traficante@unicatt.it

**Keywords:** telerehabilitation, specific learning disorders, cerebral palsy, well-being

## Abstract

Social distancing due to the COVID-19 pandemic represented a golden opportunity to implement telerehabilitation for clinical groups of children. The present study aims to show the impact that telerehabilitation had on the experience of well-being of children with special needs being treated at the Foundation IRCCS Neurological Institute ‘C. Besta’ in Milan (Specific Learning Disorders and Cerebral Palsy diagnosis); it aims to do so by comparing it with experiences of those who did not undertake telerehabilitation despite the diagnosis during the pandemic, and with typically developing children. Results show that the three groups differed in the Support, Respect and Learning dimensions of well-being experience. Post hoc comparisons revealed that children with Specific Learning Disorders and Cerebral Palsy scored higher than normotypical children in Support and in Respect scales. Furthermore, children who experienced telerehabilitation showed the highest scores on the Learning scale in comparison with the other two groups. These results support the importance of reorganizing care and assistance by integrating telemedicine, which seems to have fostered a positive experience of well-being in people with special needs, particularly in the perception of a supportive environment that respects psychological needs.

## 1. Introduction

The COVID-19 pandemic crisis is causing concern for the health and well-being of the world as a whole. Social distancing, in response to the pandemic, has also implied social isolation which has exposed, especially children, to the risk of developing relational distress and particularly, in the clinical setting, in the case of those children who were following a rehabilitation program related to their diagnosis that they have, in many cases, interrupted. The COVID-19 pandemic has intensely affected Italy, in particular Lombardy and Milan, since March 2020; among the measures implemented at national and local level to reduce the contagion, there has been the reorganization of hospitals in general. In particular, the Foundation IRCCS Neurological Institute ‘C. Besta’ in Milan, which had initially been conceived as a hub for non-COVID patients with neurological pathologies, in order to ensure continuity of care for all patients already followed, has reorganized and converted its activities to remote [1]. Specifically for children, this decision forced specialists to temporarily suspend all the rehabilitation protocols [2,3] necessitating alternative methods to guarantee, initially at least, the supervision of regular home exercises and activities for children with different types of special needs.

In Italy, child neuropsychiatric services were obliged to interrupt care assistance due to regulations provided by the government as measures for the containment of the virus (i.e., “Io resto a casa”—“I’m staying home” [4]). Distinct ways were followed in order to conform to these regulations: smart working, wage compensation (funds), forced holidays, part-time work. Measures such as working remotely and assured telepractice compensation from the public health system were supported by a regional decree within the Lombardy region, where Milan is the principal city, which was amongst the most affected regions. Indeed, the Italian Society of Infantile Neuropsychiatry (SINPIA) drafted a practical document about the service reorganization, highlighting the issues of important decrees for the Neuropsychiatry for Children. An important topic and worldwide line of research arose from the need to re-organize e-health clinical activities in order to ensure continuity of care for both adult and pediatric patients [5]. The Italian Institute of Health (ISS—Istituto Superiore di Sanità) has created a document [6] defining the rules and types of clinical activities within telemedicine, also including children and adolescents with special needs [7]. Moreover, international and national scientific associations have drawn up guidelines for the use of telemedicine in clinical developmental psychology and neuropsychology [8,9].

The attention provided by both clinicians and the world of research has also highlighted the risks of the pandemic emergency concerning the maintenance of human rights for children with disabilities and in poor socioeconomic conditions and disability [10]. However, there were already published studies on the different possible telerehabilitation interventions in different clinical populations available during the pre-COVID period [11]; these studies were valuable in guiding the choice of telerehabilitation courses for children with special needs, such as Cerebral Palsy, Language Disorders, Intellectual Disability, Developmental Learning Disorders. During the COVID-19 pandemic period, regarding telemedicine and in particular telerehabilitation in the pediatric age, several positive experiences are already present, some of which involve methods already in progress pre-COVID and others forcedly initiated due to the lockdown [12].

Moreover, at the Foundation IRCCS Neurological Institute ‘C. Besta’, the COVID-19 pandemic has provided new opportunities for rehabilitation of children with special needs, as children with Cerebral Palsy (CP) and children with Specific Learning Disorders (SLD) have benefited from the comprehensive integration of environment and family, in line with the International Classification of Functioning, Disability and Health (ICF) psychosocial model [10].

The reorganization of rehabilitation was not intended as a simple transfer of remote therapeutic paths; instead, it required the redefinition of objectives and rehabilitation methods, with the maintenance of the same therapist. In order to revise the therapeutic protocols with respect to telemedicine, the specialists verified the possibility of a child telerehabilitation basing on an informative checklist, which includes: child’s characteristics (age, diagnosis, level of severity, physical and sensory aspects, cognitive-behavioral profile, communicative and linguistic skills); family compliance (access to technological device, i.e., laptop, tablet and software, adequate space at home, parents’ level of collaboration, pending e-learning requests). Then, together with the parents, a new care and rehabilitation plan is developed. Several clinical centres that experimented with telemedicine and telerehabilitation pathways during the COVID-19 lockdown (first phase) have carried out research on the perception and satisfaction of stakeholders in telerehabilitation [3,13]. During the first phase of the pandemic (from March to September 2020 period) Pareyson and colleagues [1] administered a big survey to parents, caregivers and patients of the Foundation IRCCS Neurological Institute ‘C. Besta’ in Milan and showed that satisfaction about the telehealth and telerehabilitation was very high.

If the above underlines the impact of the pandemic on rehabilitation and care institutions, at the level of the individual, the social distancing due the pandemic undoubtedly represented a challenge also for well-being and emotional experiences for children and adolescents. This scenario led to children’s perception of a feeling of isolation and lack of relational support with effect on the emotional and well-being experience, especially due to the absence of the scholastic environment [14]. From a social and ecological perspective, schools can be considered as environments in which multifaceted interactions occur at the environmental, organizational and individual level [15], together with the effect of these different levels of interactions on individual and collective health and well-being. This is not surprising, since the impact of the school context on well-being is well recognized by recent literature [16,17,18]. In this vein, psychological well-being is understood and studied by adopting the positive psychology perspective, namely conditions and processes that contribute to the optimal functioning of individuals, groups, and institutions [19]. Current literature begins to explore, in a multidimensional way, areas such as evolution and predictors of happiness, subjective well-being, optimism, self-determination, creativity, talent and positive youth development [20,21,22,23,24,25]; this is in line with the construct of flourishing [26,27,28], which extends beyond the exclusive focus on psychopathology [29]. A focus on flourishing is particularly important because childhood and adolescence are pivotal stages of development that carry lifetime implications for functioning.

The impact that COVID-19 pandemic had on the well-being of typically developing children and adolescents was immediately a research object in the most affected countries, starting with China, and then in other countries. In particular, alarming levels of behavioral and emotional disturbances in children have been detected [30,31]. The state of mental health within the pediatric population has been studied in relation to the measures adapted to contain the virus, initially analyzing the social isolation and sense of loneliness experienced [32]. This research presented revelations using investigations oriented to the psychological, emotional and social consequences of children and adolescents.

The psychological consequences of the lockdown due to the pandemic were primarily studied on caregivers, who were stressed by the fear of contagion, the activation of smart working, job losses and consequently the effects this stress had on their children. Parental self-efficacy seems to play an important role in moderating the consequences of parental distress on children’s well-being [13]. Anti-contagion measures have involved not only all extracurricular activities but also schools with the start of distance learning; indeed, the consequences on children’s cognitive and cultural development and well-being have already become the focus of numerous studies. Recommendations have been drawn up by scientific and political institutions to prevent educational inequalities and to propose distance learning during school closures [33,34,35].

What about children with special needs? Research on the impact caused by the pandemic on well-being and mental health in children with different types of special needs has been conducted through the administration of questionnaires aimed for parents, such as Child Behavior Checklist for ages 1.5–5 and for ages 4–18 [36], or questionnaires built ad hoc [37]. The most studied clinical conditions were autism, attention deficit hyperactivity disorder, obsessive compulsive disorder, disadvantaged socioeconomic status and the studies about these conditions shows the differences in emotional and behavioral profiles of children emerged on the basis of children ages, type of neurodevelopmental disorders or neurological pathology, pre-existing emotional problems, environmental factors [9,38,39]. However, to the best of our knowledge, well-being surveys have mostly involved parents, and few have involved children and adolescents.

The present study aims to show the perceived experience of well-being of children with special needs that had been through telerehabilitation during the pandemic period by comparing it with experiences of those who did not undertake telerehabilitation during the pandemic, and with typically developing children. We proposed to ask both typically developing children, and children with special needs for their direct opinion on their wellbeing experience. The purpose of our research is to verify the hypothesis that those who had a rehabilitation path pre-COVID and had the possibility to continue the rehabilitation process and care remotely during the emergency period, may have experienced greater well-being compared to those who had not benefited from it. Comparison with a normotypical group gives us the opportunity to consider the pandemic variable and control it. The implemented contagion containment measures, have, in fact, required an enormous adaptive effort from the entire pediatric population, greatly limiting the possibility of social relationships, the possibility of learning through direct experiences, involvement and satisfaction in the learning process, trust in future and quality of life. The emotional costs paid by children have been extremely high: caregivers, educational facilities, schools and families, who were stressed and afraid, have struggled to understand and satisfy the development needs of each child. Therefore, we expect that participants with special needs who have had a presumably better response from the environment in terms of continuity in care, rehabilitation and relationships with specialists may have had a better experience of well-being than those who have not received it.

## 2. Materials and Methods

### 2.1. Participants

In terms of participants, 56 children with different types of special needs were recruited from the clinical service of Developmental Neurology Unit of Foundation IRCCS Neurological Institute ‘C. Besta’ (36 children with Specific Learning Disorders and 20 children with Cerebral Palsy), and 30 normotypical children attending primary and secondary schools in Milan. All participants completed the online questionnaire, which was sent through an online survey (Google Forms) after collecting written consents by their parents, in the period from May to August 2020. All participants were native Italian speakers. The clinical study sample consisted of 36 children with Specific Learning Disorders (SLD) and 20 children with Cerebral Palsy (CP). From these two groups, children with SLD and CP who were telerehabilitation were selected for each group, respectively. These children were matched by gender, age and comorbidity in the SLD case and by age, gender and severity in the CP case with children who did not undergo telerehabilitation during the study period. More specifically, the SLD Telerehabilitation group (N = 8) had these clinical characteristics: 1 child with Dyscalculia, 2 with Dyslexia and Dysorthography, 5 with Dyslexia, Dysorthography and Dyscalculia. This group was matched with the children with SLD No telerehabilitation group (N = 8) that had the same clinical characteristics: 1 child with Dyscalculia, 2 with Dyslexia and Dysorthography, 5 with Dyslexia, Dysorthography and Dyscalculia.

For children with CP, Telerehabilitation group (N = 9) had these clinical characteristics on the base of the Classification Systems for children with Cerebral Palsy, the Gross Motor Function Classification System (GMFCS, [40]), the Manual Ability Classification System (MACS, [41]), Visual Function Classification System (VFCS, [42]): 2 children with Tetraplegia, with performances on the VFSC, GMSC and MACS between levels III and IV, with needs of substantial environmental adjustments; 4 children with Hemiplegia, with performances on VFSC, GMSC, MACS between levels I and II, with good autonomy; 3 children with Diplegia, with performances on VFSC, GMSC, MACS between levels II and III, with mild functional limitations, that need of some environmental adjustments. CP children in No telerehabilitation group (N = 9) had the same clinical characteristics: 2 subjects with Tetraplegia, 4 subjects with Hemiplegia, 3 subjects with Diplegia with clinical characteristics similar to those of the telerehabilitation group.

As for children with SLD, Telerehabilitation group (N = 8, mean age = 126.63 months; SD = 7.84 months; range min 121 months–max 145 months), had the opportunity to have 2 online treatment sessions a week (45 min), focused on reading, writing and math skills whereas children in No telerehabilitation group (N = 8, mean age = 126.62 months; SD = 7.8 months; range min 121 months– max 145 months) had finished their treatment before the pandemic period, after reaching the goals of their rehabilitation projects. More specifically they are involved in telerehabilitation with online dyslexia platform as RIDInet with Reading Trainer app and Rhythmic Reading Training RRT teleintervention [43]. The telerehabilitation used RIDInet, an internet platform that enhances reading speed and accuracy, spelling skills, text comprehension, arithmetic and numerical skills, executive functions (i.e., inhibition, working memory, cognitive flexibility), language (rapid naming, expressive skills). The Rhythmic Reading Training [43], a computer-assisted training, was designed to implement a treatment which combines a traditional approach (sublexical treatment) with rhythm processing training, and it was possible to transfer in a fairly simple and effective way remotely through the “Share screen and system audio” option present in the platforms in use the rhythmic exercises proposed.

As for children with CP, Telerehabilitation group (N = 9, mean age = 131.37 months; SD = 24.16 months; range min 93 months–max 173 months) was supported by 1 online treatment sessions (45 min), for mean 13 weeks, tailored on their neuro-psychomotor needs, whereas children in the No telerehabilitation group (N = 9, mean age = 132 months; SD = 27.8 months; range min 85 months–max 174 months) did not show, in that period, any specific need to be addressed through rehabilitation projects. Tele-treatment for children with CP consisted of real-time treatment for children with a neuropsychological and learning exercise program; sharing of either information with parents and with special need school teachers or an exercise program to be implemented by the parents or teachers. For these children with motor needs, video-tutorials were sent to parents twice a week, in which the exercises to be performed by the child were explained. The weekly supervised meeting with the therapist allowed parents to be correctly guided and above all, it allowed for subjects to maintain contact with the therapist; giving information and technical feedback about pharmacological treatment, checking the effects of the therapy on motor pattern and adaptive functions. Furthermore, two children continue the work about the study method that aimed to integrate compensatory tools in combination with the results of his CP (use of speech already started in presence with the aim of producing texts and implementing corrections independently).

All children were tested to verify the efficacy of the treatment at the end of the rehabilitation cycle. In all, a benefit was found in their performances and instrumental skills. As for the normotypical children (N = 30), recruited from school in Milan, they had no diagnosis of special needs. They were matched to the clinical groups by gender and age (see Table 1 for the descriptive statistics).

In sum, in order to assess the impact of telerehabilitation on well-being in children with special needs, three groups were selected for both SLD and CP conditions: children with SLD or CP who made experience of telerehabilitation (N = 17; F = 8), children with SLD or CP who did not experience telerehabilitation (N = 17; F = 6), normotypical children (N = 17; F = 6). They were matched by gender (χ^2^ = 1.416, *p* = 0.923) and age (see Table 1; F_group_ = 1.47, *p* = 0.232; F_telerehabilitation_ = 0.011; F_interaction_ = 0.189).

### 2.2. Measures

Two questionnaires were administered to assess children’s wellbeing.

#### 2.2.1. Comprehensive Inventory of Thriving for Children

Comprehensive Inventory of Thriving for children [44] consists of a comprehensive range of subscales for assessing one facet of psychological well-being each, with three items. The Italian adaptation of the CIT to child population has 45 items assessing 15 facets of positive functioning, representing the dimensions of psychological well-being: Support (e.g., There are people who give me support and encouragement); Respect (e.g., People are polite to me); Loneliness (e.g., Often I feel left out); Belonging (e.g., I feel a sense of belonging in my community); Engagement (e.g., In most of the things I do, I feel energized); Skills (e.g., I get to do what I am good at every day); Learning (e.g., Learning new things is important to me); Self-worth (e.g., What I do in life is valuable and worthwhile); Optimism (e.g., I have a positive outlook on life); Life satisfaction (e.g., My life is going well), Positive feelings (e.g., I feel happy most of the time); Negative feelings (e.g., I feel bad most of the time). Cronbach’s alpha for the total scale is 0.86.

#### 2.2.2. Scale of Positive and Negative Experience

The Scale of Positive and Negative Experience (SPANE) [45] is a brief 12-item scale, with six items devoted to positive experiences (items: positive, good, pleasant, happy, joyful, and contented) and six items designed to assess negative experiences (items: negative, bad, unpleasant, sad, afraid, and angry). The scale is aimed to assess the full range of positive and negative experiences: it not only assesses the pleasant and unpleasant emotional feelings, but also reflects other states such as interest, flow, positive engagement, and physical pleasure. Each SPANE item is scored on a scale ranging from 1 to 5, where 1 represents ‘‘very rarely or never’’ and 5 represents “very often or always”. Moreover, the scale is keyed to the last 4 weeks, which is short enough to allow the respondent to recall actual experiences rather than rely on general self-concept [45]. The positive and negative scales are scored separately because of the partial independence of the two types of feelings. The summed positive score (SPANE-P) can range from 6 to 30, and the negative scale (SPANE-N) has the same range. The scale has been translated into several languages, including Italian and can be downloaded for research purposes on the official website of the authors the scale has been translated into several languages, including Italian and can be downloaded for research purposes on the official website of the authors. The analysis conducted on 407 Italian children [44] showed good alpha coefficients (SPANE-P: α = 70; SPANE-N: α = 67).

### 2.3. Procedure

An invitation to participate in the research was sent to all families whose children were in charge of Developmental Neurology Unit, Foundation IRCCS Neurological Institute ‘C. Besta’ of Milan. The rate of positive responses was 92%. Parents who gave their consent communicated to the researchers the email address to be used to get in contact with their children. Each participant received, by email, a link to fill in the questionnaires on the online survey platform. Questionnaires were administered by researchers and were completed individually by children. Data collection lasted from May 2020 to August 2020.

### 2.4. Statistical Analyses

(1) In order to assess the differences in CIT-C scales between groups (Telerehabilitation, No telerehabilitation, Normotypical control), a MANOVA was carried out on the CIT-C scales raw scores with Group (Telerehabilitation, No telerehabilitation, Normotypical control) and Condition (SLD, CP) as independent variables;

(2) The effects of Group and Condition on SPANE scores (Positive and Negative) were considered as independent factors in two ANOVAs.

Post hoc comparisons were applied to analyze significant differences in detail.

## 3. Results

First, it is worth noting that all three groups of children (Telerehabilitation, No telerehabilitation, Normotypical control) showed mean scores on CIT-C scales within 1.5 SD from the normative mean (Table 1).

Results from MANOVA showed that the main effect of Group was significant (Group Pillai’s trace: *F*_24, 70_ = 3.14, *p* < 001; *η^2^* = 0.518), irrespective to the Condition, which did not reach significance level neither as main factor (Pillai’s trace: *F*_12, 34_ = 1.43, *p* = 201; *η^2^* = 0.335), nor in interaction with Group (Pillai’s trace: *F*_24, 70_ = 1.51, *p* = 095; *η^2^* = 0.34). One-way ANOVAs showed that the three groups differed on Support (*F*_2, 45_ = 7.17, *p* = 002; *η^2^* = 0.242), Respect (*F*_2, 45_ = 3.43, *p* = 041; *η^2^* = 0.132) and Learning (*F*_2, 45_ = 7.29, *p* = 002; *η^2^* = 0.245) scales. In post hoc comparisons, Sidak adjusted (Table 2) revealed that children with SLD and CP (irrespective of the experience of Telerehabilitation) scored higher than normotypical children on the Support and in Respect scales. Children with SLD and CP, who experienced telerehabilitation, showed the highest scores on the Learning scale, in comparison with the other two groups.

ANOVAs carried out on SPANE scores did not reveal any significant difference neither by Group nor by Condition (Table 3).

## 4. Discussion

Our research goal was to determine the effects of telerehabilitation during the pandemic period on the well-being experienced by children and adolescents with special needs. This was assessed by comparing the experiences of those who received telerehabilitation with those who did not receive it, and with typically developing children. To the best of our knowledge, this is the first study that compared the well-being of children with special needs and typical development during the lockdown phase of COVID-19 pandemic, which investigated the children’s direct opinions. The questionnaires explored the psychological well-being of children from a multidimensional perspective [44,46]. Regarding these dimensions, our results reveal that children with SLD and CP, independently from the Telerehabilitation experience, scored higher on the relational dimension (support and respect scales) compared to the normotypical group. In addition, children with Specific Learning Disorders and Cerebral Palsy, who experienced telerehabilitation, showed the highest scores in Learning dimension, in comparison with the other two groups. Non-significant results are shown about the perception of negative and positive feelings.

Starting from the significant difference in Learning, the dimension between children with special needs who were in telerehabilitation during the pandemic period and children with special needs who did not is described. The Learning subscale requires to express the statement such as “new things are important to me”, “I learned something new yesterday”, “I always learn something every day” and it concerns a sense of mastery and accomplishment. In other words, we asked the children to answer, during the pandemic crisis, what had become of their motivation/interest in learning new subjects after being forced to stay home from school due to social distancing. In this way, the children responded about the impact and role of the environment on the pupils’ intellectual curiosity. In a period of confinement due to the COVID-19 pandemic, in which remote learning has not always been reorganized in a timely manner, the perception of having learned ‘new things’ may have generally been limited in students. Children with special needs may have experienced, even more, the impossibility of accessing normal school learning paths and the difficult reorganization of the remote learning system, besides the development of high levels of stress/anxiety and emotional distress, in addition to low levels of well-being, self-esteem and self-efficacy [47]. The possibility for some of them to remotely continue the telerehabilitation might have influenced the well-being component linked to cultural and personal enrichment, unlike subjects with special needs who have not sustained telerehabilitation and subjects with typical development. These results help to shed a light on the role of telerehabilitation on the well-being of children with special needs. Conversely, some studies about the Specific Learning Disorders had already shown during the pre-COVID period the positive impact on the motivation of students who use new technologies [11]. Furthermore, another aspect concerns curiosity of children about the use of technological devices and its integration in the rehabilitation, which was usually carried out in person. They did not report difficulties in the use of new technologies. The one-to-one interaction and the continuous and complete attention to the child have contributed to making the intervention more dynamic and engaging despite being remotely conducted. However, it must be highlighted that remote activity requires commitment and responsibility on behalf of the child in order for it to be successful, and children therefore proved to be more active, more motivated and more responsible. The family environment setting was a strong point, also becoming a starting point for conversation. In this study, children who benefited from telerehabilitation might have felt they are improving and are generally more engaged, which may have positive impacts and effects on their well-being.

Regarding the result of the relationship dimension, our analysis shows that children with special needs, regardless of whether or not telerehabilitation has taken place, reported higher scores than children with typical development on the Respect and Support scales; these concern the perceived well-being of supportive and enriching relationships, and the environmental response component therefore appears as an important factor of well-being. The response of the Developmental Neurology Unit of Foundation IRCCS Neurological Institute ‘C. Besta’ of Milan since the beginning of the lockdown and the main objective of the distance reorganization, was precisely to ensure the continuity of care to patients under treatment and the provision of a prompt support response. Children with special needs in the present study were all patients already known to the specialists, with management consisting of periodic follow-ups, subsequently converted into distance sessions, and some of them were included in rehabilitation programs. For example, in line with national and international guidelines, the treatment (regular care) for children with CP is extensive, 2–3 times per week up to 6 years of age; for older aged children, depending on the objectives, the frequency of treatment is reduced (1–2 times per week), while extensive treatments are carried out in severe neuropsychological or learning disorders, and specific focus-intensive cycles, 2–3 times per year. The results of this study, even with limitations, contribute to the literature confirming that telemedicine reorganization provides a comfortable environment for young patients. Moreover, in our study, all children had already been diagnosed in our institute, although not all of them were undergoing rehabilitation. Previous research has investigated the experience of well-being in children and adolescents with special needs; indeed, the presence of neurodevelopmental disorders can be a risk factor for the development of emotional and behavioral problems, and not only has an impact on learning possibilities but also on well-being. However, some studies with students with learning difficulties show that having a diagnosis seems to have a protective role for their psychological well-being compared to those children who, despite presenting difficulties, have never received a diagnosis [17]. Recently, Lombardi and colleagues (2021) underlined the importance of receiving a diagnosis, as it seems to function as a protective agent for students’ psychological and scholastic well-being. Since the two groups of children with special needs (with and without telerehabilitation) faced similar positive well-being experiences, with only one benefit in the Learning subscale in the telerehabilitation condition, it can be explained by considering the possible “costs” of telerehabilitation itself. In a difficult period for Italian families, in which parents were often engaged in smart working and distressed by feelings of fear and uncertainty, the offer of telerehabilitation—and, in general, continuity of care and assistance—may have granted children with the attention they needed from the adult world and thus filled a “void”. Indeed, it is important to promote the benefits but also to assume that the commitments of telerehabilitation are often added to those of distance learning, and this may have had an impact on the well-being of those who maintained rehabilitation through telemedicine. Finally, it is important to emphasize that the three groups of subjects (Telerehabilitation, No telerehabilitation, Normotypical control) reported mean scores within 1.5 Standard Deviation from the normative mean. Furthermore, many children with special needs showed improved autonomy, and some children showed improved learning and attention during the home confinement, as confirmed by teachers. This may in part be explained by the increased time spent at home with their family or perhaps improved motivation resulting from the reduced effort and time involved in school and, for children with special needs, rehabilitation activities. It should also be noted that, with regards to the Italian adult population, [48] revealed that 38% of the general population during the COVID-19 pandemic perceived a form of psychological distress but the majority of subjects displayed no relevant distress. The timelines and quality of the reorganization in telemedicine partially filled, for some children, schools’ shortcomings, which had interrupted in presence lessons and, especially in the initial phase of the pandemic, had difficulty in meeting the demands of children with special needs; this manifested as online teaching organized as lessons, whereby unpreparedness and slowness in dealing with the emergency occured.

The result regarding the positive and negative experience measured by the Scale of Positive and Negative Experience (SPANE) did not reveal any significant difference neither by Group nor by Condition. This result can be explained by the same situations and the same feelings shared by all children. The impact of the restrictions due to the pandemic involved the whole world and led to shared feelings, regardless of condition. This has led to children answering questions such as “in the last 4 weeks I have felt sad” or “in the last 4 weeks I have felt happy” in the same way, regardless of whether they are in telerehabilitation or a typically developing child.

In conclusion, our findings support the importance of having reorganized care and assistance by integrating telemedicine, which may have fostered a positive experience of well-being in people with special needs, particularly in the perception of a supportive environment that respects psychological needs. The aim was to ensure continuity of care, while remaining aware that this could not replace in-person interventions. Home-based rehabilitation is an emerging feature in the literature and the difficulty, which has not always been overcome, has been that of transferring the complex regimes based on motor learning principles and psychological consultation to the home setting. The possibility of maintaining remote rehabilitation also seems to have nurtured feelings of mastery and accomplishment in children with special needs. We would therefore like to see the maintenance of hybrid methods, integrating telemedicine into clinical practice, adapted to the needs of each individual and with attention to the psychological well-being of young patients.

## 5. Limitations

Although the results of our study give some insights about the opportunity for telerehabilitation to support students’ well-being, some limitations should be emphasized, concerning the size of the sample and the not entirely homogeneous nature of the types of treatments and clinical profiles. A larger sample size would have allowed us to consider the different types of treatment performed and also to analyze the relationships and comparisons between the different variables through more complex statistical techniques, which would allow for a greater generalization of results. This was also due to the period in which the data were collected, and the initial phase of the reorganization, which prompted an investigation into these issues, regardless. However, to the best of our knowledge, this is the first study to ask children with special needs how they were approaching this period of difficulty using a control group with typical development. Therefore, we hope that the present study might be considered a “small part” of the literature on the topic of telemedicine with a focus on its effects on the well-being of young patients.

## Figures and Tables

**Table 1:** Mean age and SD (in parentheses) of the three groups by diagnosis.

Group	N	SLD	N	CP	N	Total
Telerehabilitation	8	122.5 (13.4)	9	131.37 (24.2)	17	130.35 (30.2)
No telerehabilitation	8	126.63 (7.8)	9	132 (27.8)	17	129.47 (20.5)
Normotypical control	8	125.75 (10.7)	9	131.44 (31.4)	17	128.76 (23.5)

SLD = Specific Learning Disorder children; CP = Cerebral Palsy children.

**Table 2:** CIT-C’s mean scores and SD of the three groups by diagnosis (significant differences in bold).

CIT-C Scale	Group	SLD		CP		Total	
		M	SD	M	SD	M	SD
Support	Telerehabilitation	4.46	0.5	4.89	0.2	**4.73**	0.3
	No telerehabilitation	4.71	0.3	4.74	0.4	**4.69**	0.4
	Normotypical control	4.17	1.0	3.89	0.8	**4.02**	0.9
Respect	Telerehabilitation	3.88	0.6	4.74	0.4	**4.33**	0.7
	No telerehabilitation	4.17	1.1	4.59	0.8	**4.39**	0.9
	Normotypical control	3.63	0.7	3.78	1.1	**3.71**	0.9
Loneliness	Telerehabilitation	3.50	0.5	3.52	1.0	3.51	0.8
	No telerehabilitation	4.13	0.7	3.85	0.8	3.98	0.8
	Normotypical control	4.12	0.5	3.93	1.1	4.02	0.8
Belonging	Telerehabilitation	4.29	0.9	4.44	0.9	4.37	0.9
	No telerehabilitation	4.50	0.9	4.19	1.0	4.33	0.9
	Normotypical control	4.00	0.9	3.81	0.9	3.90	0.9
Engagement	Telerehabilitation	3.21	0.8	3.89	0.9	3.57	0.9
	No telerehabilitation	3.92	0.6	3.74	0.9	3.82	0.7
	Normotypical control	3.71	0.8	2.93	0.8	3.29	0.9
Skills	Telerehabilitation	3.88	0.7	3.85	1.0	3.86	0.8
	No telerehabilitation	3.54	1.0	3.67	1.2	3.61	1.0
	Normotypical control	3.58	0.7	3.15	0.7	3.35	0.7
Learning	Telerehabilitation	4.38	0.5	4.41	0.8	**4.39**	0.7
	No telerehabilitation	3.63	0.7	3.63	0.8	**3.63**	0.8
	Normotypical control	3.79	0.8	3.30	0.4	**3.53**	0.7
Self-worth	Telerehabilitation	3.75	1.1	3.59	1.0	3.67	1.0
	No telerehabilitation	3.88	0.4	3.78	1.0	3.82	0.7
	Normotypical control	3.58	0.8	3.19	0.9	3.37	0.9
Optimism	Telerehabilitation	3.63	0.8	4.15	0.9	3.90	0.9
	No telerehabilitation	4.50	0.6	3.93	0.9	4.20	0.8
	Normotypical control	3.58	0.9	3.41	1.0	3.49	0.9
Life Satisfaction	Telerehabilitation	3.17	1.2	4.00	1.2	3.61	1.2
	No telerehabilitation	3.70	0.9	3.78	1.1	3.74	1.0
	Normotypical control	3.92	0.8	4.04	0.9	3.98	0.8
Positive feelings	Telerehabilitation	3.33	1.1	4.37	1.0	3.88	1.1
	No telerehabilitation	4.63	0.5	3.74	1.2	4.16	1.0
	Normotypical control	3.67	0.6	3.78	0.9	3.73	0.7
Negative feelings	Telerehabilitation	2.96	1.3	3.63	1.3	3.31	1.3
	No telerehabilitation	4.00	0.8	3.63	1.1	3.80	1.0
	Normotypical control	3.96	0.3	4.15	0.9	4.06	0.7

SLD = Specific Learning Disorder children; CP = Cerebral Palsy children. SPANE = Scale of Positive and Negative Experience.

**Table 3:** SPANE’s mean scores and SD of the three groups by diagnosis.

SPANE Scale	Group	SLD		CP		Total	
		M	SD	M	SD	M	SD
Negative	Telerehabilitation	16.2	2.5	15.6	7.1	15.9	5.3
	No telerehabilitation	12.1	2.4	13.7	6.1	12.9	4.7
	Normotypical control	13.5	4.6	13.4	6.1	12.2	4.4
Positive	Telerehabilitation	19.1	4.0	23.9	4.7	21.7	4.9
	No telerehabilitation	25.4	2.0	22.2	6.1	23.7	4.8
	Normotypical control	22.5	5.6	23.1	5.6	22.8	5.5

SLD = Specific Learning Disorder children; CP = Cerebral Palsy children. CIT-C = Comprehensive Inventory of Thriving for children.

## Data Availability

The data presented in this study are available on request from the authors. The data are not publicly available due to privacy restrictions.
